# Thrombotic thrombocytopenic purpura developed during the conservative treatment of anti-phospholipase A_2_ receptor antibody-positive idiopathic membranous nephropathy: a case report

**DOI:** 10.1186/s12882-020-02086-z

**Published:** 2020-10-12

**Authors:** Rei Iio, Shin’ichi Akiyama, Kensuke Mitsumoto, Yukimasa Iwata, Hiroki Okushima, Karin Shimada, Naomi Ota, Kodo Tomida, Hiroaki Fushimi, Tatsuya Shoji, Masanori Matsumoto, Terumasa Hayashi

**Affiliations:** 1 Department of Kidney Disease and Hypertension, Osaka General Medical Center, 3-1-56 Bandaihigashi, Sumiyoshi-ku, Osaka, 558-8558 Japan; 2 Division of Nephrology, Department of Internal Medicine, Nagoya University Graduate School of Medicine, 65 Tsurumai-cho, Showa-ku, Nagoya, 466-8550 Japan; 3 Department of Pathology, Osaka General Medical Center, 3-1-56 Bandaihigashi, Sumiyoshi-ku, Osaka, 558-8558 Japan; 4 Department of Blood Transfusion Medicine, Nara Medical University, 840 Shijyo-cho, Kashihara, Nara 634-8522 Japan

**Keywords:** Anti-PLA_2_R antibody, Idiopathic membranous nephropathy, ADAMTS13 inhibitor, Acquired thrombotic thrombocytopenic purpura

## Abstract

**Background:**

Idiopathic membranous nephropathy (MN) is one of the major glomerulonephritis that cause nephrotic syndrome. The phospholipase A_2_ receptor (PLA_2_R) has recently been identified as an endogenous antigen of idiopathic MN. Thrombotic thrombocytopenic purpura (TTP) is a disorder characterized by schistocytes, hemolytic anemia, thrombocytopenia, and organ dysfunction which occurs as a result of thrombi. Patients with acquired TTP have autoantibodies against a disintegrin and metalloprotease with thrombospondin type 1 motif 13 (ADAMTS13). These autoantibodies act as an inhibitor and cause ADAMTS13 deficiency. Idiopathic MN and acquired TTP are usually considered as independent autoimmune diseases. We experienced a patient who developed TTP during the conservative treatment of idiopathic MN, with the coexistence of ADAMTS13 inhibitor and anti-PLA_2_R antibody.

**Case presentation:**

A 73-year-old man presented with thrombocytopenia, hemolytic anemia, disturbance of consciousness, and acute kidney injury after 4-year course of biopsy-proven idiopathic MN. ADAMTS13 activity was undetectable and the ADAMTS13 inhibitor was identified. Additionally, he was positive for anti-PLA_2_R antibody. The patient did not have any diseases that could cause secondary thrombotic microangiopathy, and he was diagnosed with acquired TTP. Steroid therapy and plasma exchange were initiated and the acquired TTP resolved. MN achieved remission 3 months after the anti-PLA_2_R antibody disappeared.

**Conclusions:**

This is the first reported case of acquired TTP developed during conservative treatment of idiopathic MN, with both ADAMTS13 inhibitor and anti-PLA_2_R antibody positive at the onset of the TTP. The present case suggests that idiopathic MN might be associated with the development of some cases of acquired TTP.

## Background

Membranous nephropathy (MN) is a kidney disease that often causes nephrotic syndrome. MN is roughly classified into 2 types: idiopathic MN, which develops without any underlying disease, and secondary MN, which develops due to collagen disease, malignant disease, infection, or drug use. The phospholipase A_2_ receptor (PLA_2_R) and thrombospondin type-I domain-containing 7A (THSD7A) have been identified as endogenous antigens of idiopathic MN [1, 2]. Additionally, it has been reported that serum anti-PLA_2_R antibody levels are associated with disease activity, such as the level of proteinuria, and the therapeutic response in idiopathic MN [3]. Therefore, the serum anti-PLA_2_R antibody level can be used in the diagnosis of idiopathic MN.

Thrombotic thrombocytopenic purpura (TTP) is a disorder characterized by schistocytes, hemolytic anemia, thrombocytopenia, and organ dysfunction caused by thrombi. In terms of renal pathology, the microscopic features of TTP are the same as those of thrombotic microangiopathy, with ectatic glomerular capillary lumina, enlargement of the subendothelial space, mesangiolysis, and reduplication of the glomerular capillary basement membranes. Recent studies have shown that TTP is caused by a deficiency in a disintegrin and metalloprotease with thrombospondin type 1 motif 13 (ADAMTS13), which cleaves von Willebrand factor and prevents excessive platelet aggregation. Patients with acquired TTP have autoantibodies against ADAMTS13, causing a deficiency of ADAMTS13. As a result, platelet thrombi are formed in the microvessels of multiple organs.

Idiopathic MN and acquired TTP are usually regarded as independent diseases. However, herein, we report a case of acquired TTP developed 4 years after the onset of idiopathic MN. In our case, in response to plasma exchange and steroid therapy, both the anti-PLA_2_R antibody level and ADAMTS13 inhibitor level became undetectable, and these 2 diseases resolved.

## Case presentation

**Fig.1:**
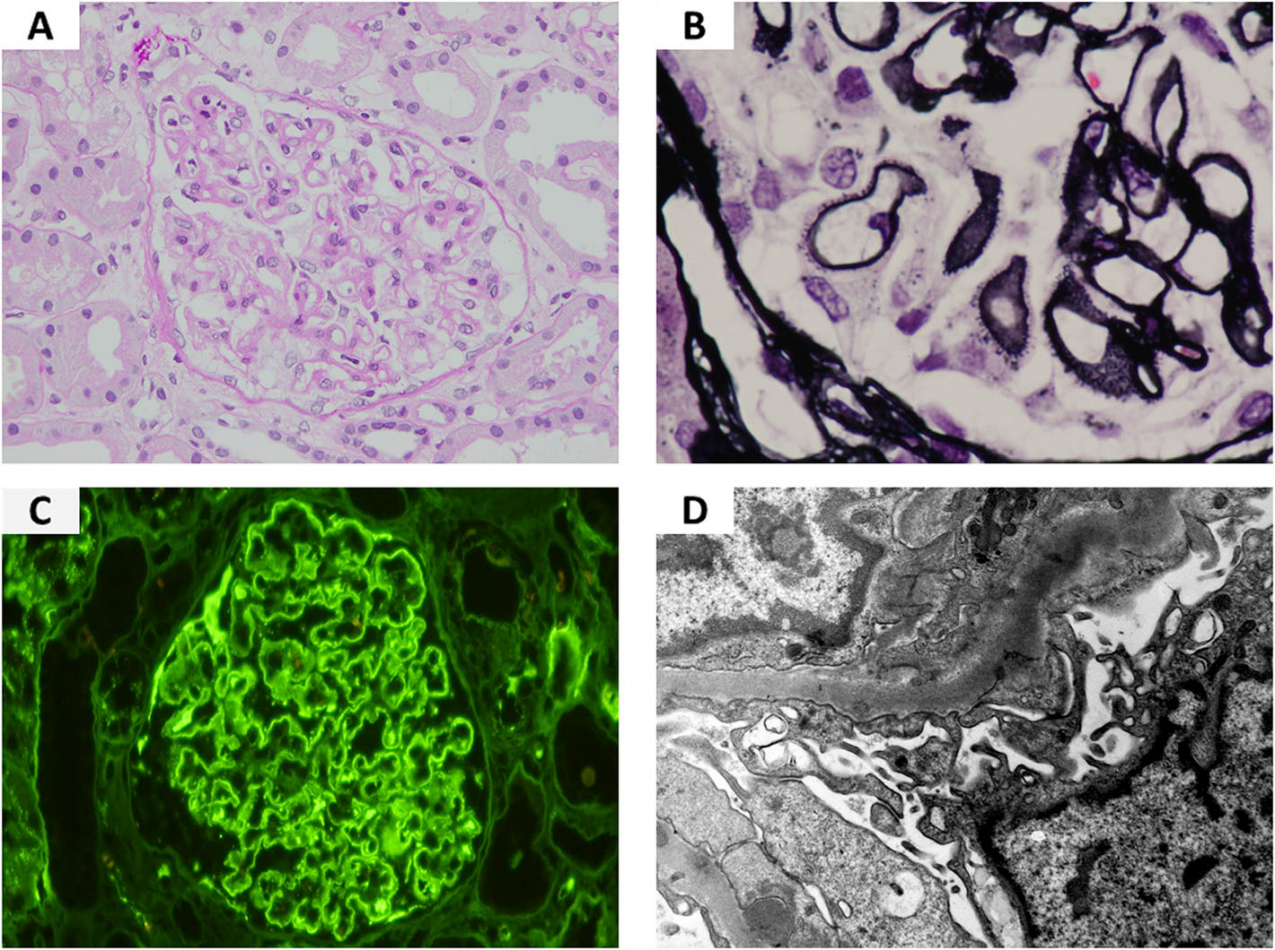
Histopathological findings in the kidney biopsy from the patient. (**a**): A glomerulus with thickened basement membranes and normal cellularity is shown (periodic acid-Schiff stain, × 200) (**b**): A glomerulus with a bubbly appearance and spike formation of the glomerular capillary walls is shown (periodic acid silver-methenamine stain, × 400). (**c**): Intense immunofluorescence staining for immunoglobulin G on the glomerular capillary walls is shown (× 200) (**d**): Subepithelial electron-dense deposits and effacement of the podocyte foot processes are observed on electron microcopy (× 8000)

A 69-year-old man was referred to our department with proteinuria and edema of the lower limbs in July 2012. His urinary protein excretion was 7.8 g/day and serum creatinine level was 2.10 mg/dL. He was diagnosed with nephrotic syndrome, and a renal biopsy was performed. Light microscopy showed glomerular capillary thickening with periodic acid-Schiff staining (Fig. 1a), and a bubbly appearance and spike formation in the glomerular capillary walls, with periodic acid silver-methenamine staining (Fig. 1b). Immunofluorescence staining showed granular 2 + deposition of immunoglobulin G and complement C3 in the glomerular capillary walls (Fig. 1c). Electron microscopy demonstrated subepithelial deposits. MN was diagnosed as stage II (Ehrenreich-Churg classification) (Fig. 1d). Examinations for collagen disease and malignancy were performed and were negative. Additionally, he did not use any drugs that could cause nephrotic syndrome. Based on these results, he was diagnosed with idiopathic MN.

During hospitalization, paroxysmal atrial fibrillation occurred, and oral warfarin was started. Steroid therapy was initiated for MN, and complete remission was achieved. However, the nephrotic syndrome relapsed while the patient was on prednisolone (PSL) at a dose of 5 mg per day. As hypoalbuminemia and edema were manageable by conservative treatment, the dosage of the steroid was maintained, and 2.5 years after the onset of MN, steroid therapy was discontinued.

Four years after his MN diagnosis, he lost consciousness at home, and he was subsequently admitted to our hospital. His consciousness level on the Glasgow Coma Scale was E4V2M5. The patient did not have a fever. His blood pressure was 60/30 mmHg. His pulse rate was 90 beats/min and irregular. Physical examination showed lower extremity edema and petechiae on his front chest. Laboratory blood data on admission were as follows; white blood cell count, 8200 /μL; hemoglobin, 4.8 g/dL; platelet count, 1.7 × 10^4^ /μL; direct bilirubin, 1.4 mg/dL; indirect bilirubin, 4.5 mg/dL; lactate dehydrogenase, 1477 IU/L; C-reactive protein, 2.85 mg/dL; fibrinogen, 275 mg/dL; and fibrin degradation products, 15.9 μg/mL. A direct Coombs test was negative, and schistocytes (4.4%) were detected. As a result, he was diagnosed with thrombotic microangiopathy (TMA).

The patient’s serum creatinine level at the latest visit was 1.83 mg/dL, and it had worsened to 3.76 mg/dL on admission. On the day after hospitalization (day 2), fever was observed, thus his medical condition was consistent with TTP. Disseminated intravascular coagulation (DIC), hemolytic uremic syndrome (HUS), and secondary TMA were considered as differential diagnoses. However, his blood, urine, and sputum cultures were negative. In addition, whole-body contrast-enhanced computed tomography revealed no cancer or a focus of infection. Although diarrhea was observed before admission, his stool culture and serum anti-lipopolysaccharide antibodies were negative, and no bloody stool was observed. Therefore, the possibility of HUS was excluded. Furthermore, he had no history with medications that could cause secondary TMA, and there were no laboratory results suggestive of collagen disease. From these results, acquired TTP was strongly suspected, and plasma exchange was started on the day of admission (day 1). On day 13, his ADAMTS13 activity was found to be undetectable (< 0.5%) and his ADAMTS13 inhibitor level was 1.2 Bethesda U/mL on the admission blood sample. Thus, he was diagnosed with acquired TTP. On the same day, 1 mg/kg (55 mg) per day of PSL was started. On day 23, we withdrew the plasma exchange because the ADAMTS13 inhibitor level became undetectable and the ADAMTS13 activity was 30.2%. On day 68, he was discharged from our hospital.

**Fig. 2:**
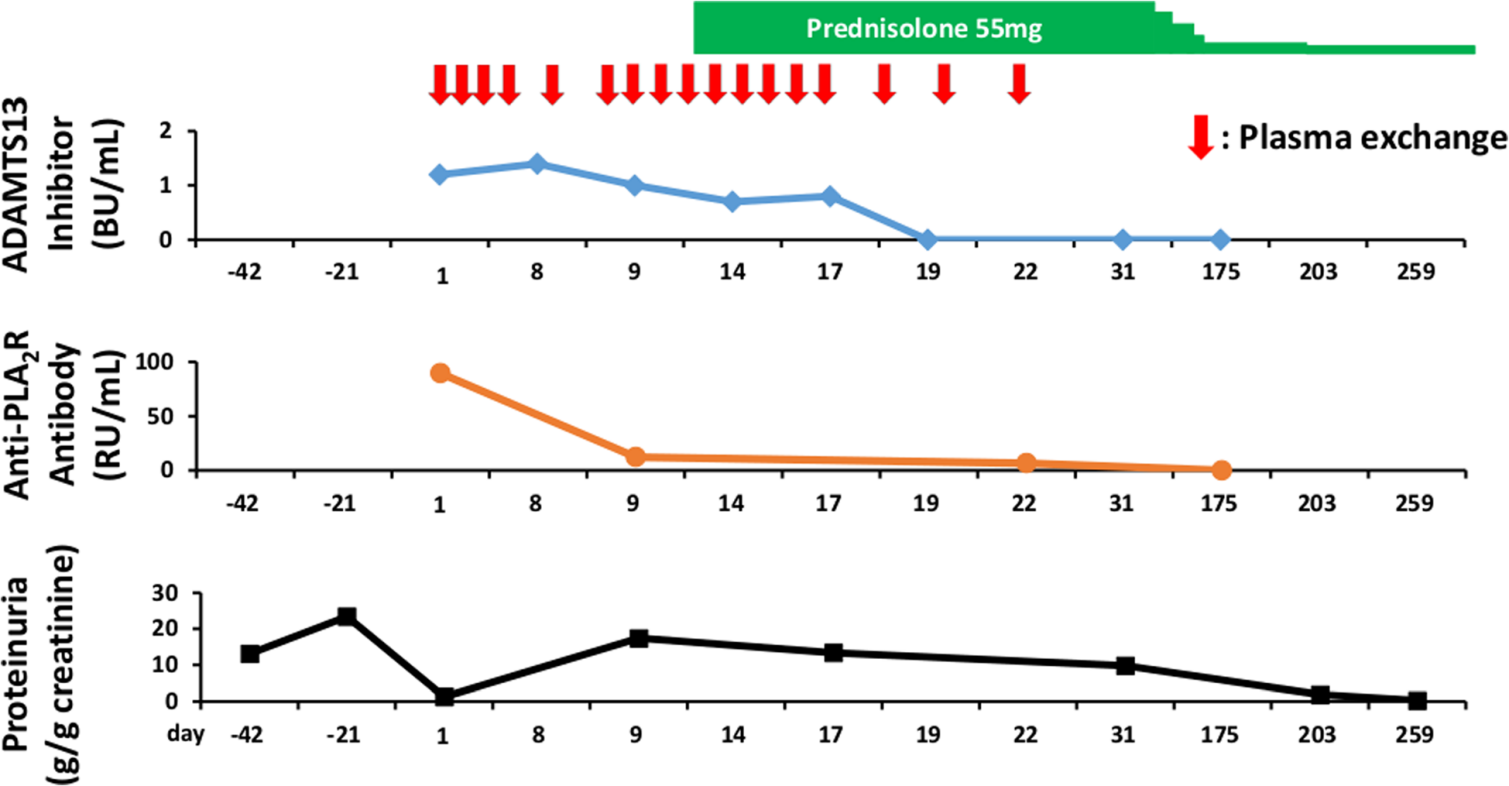
ADAMTS13 inhibitor (blue diamonds), anti-PLA_2_R antibody (orange circles), and proteinuria (black squares) concentrations during treatment with plasma exchange and steroid therapy. Day 1 is the day of admission

The urinary protein creatinine ratio (g/gCr) at the onset of TTP was 1.8 and thereafter increased to a maximum level of 26.6, then decreased to 7.9 at discharge. The anti-PLA_2_R antibody level was 89.3 RU/mL at the onset of TTP, and 6.6 RU/mL at the end of the plasma exchange. On day 175, the anti-PLA_2_R antibody level became undetectable. The ADAMTS13 activity was 23.0% and the ADAMTS13 inhibitor level kept undetectable. On day 259, complete remission of MN was achieved with 10 mg /day of PSL, and his serum creatinine level recovered to 0.96 mg/dL. Since then, there has been no recurrence of either disease (Fig. 2). Anti-THSD7A IgG level was undetectable.

## Discussion and conclusion

In the present case, the acquired TTP developed during the conservative treatment of idiopathic MN. Secondary MN and secondary TMA can be caused by collagen diseases such as systemic lupus erythematosus and Sjogren's syndrome, malignancies, and certain drugs, and can sometimes coexist [4]. In contrast, idiopathic MN and acquired TTP have different etiologies, and their coexistence is unusual. In this case, it is possible that these 2 diseases merged by chance. However, as far as our literature review, 3 cases of idiopathic MN and acquired TTP co-existing have been reported [5–7], which suggested to us a possible association between these 2 diseases. Several mechanisms which could explain this association might be proposed. First, the presence of a common epitope in PLA_2_R and ADAMTS13 may have induced the production of antibodies to ADAMTS13. The structures of PLA_2_R and ADAMTS13 have a cysteine-rich domain in common. However, the cysteine-rich domains of PLA_2_R and ADAMTS13 have low homology on Protein BLAST (National Center for Biotechnology information, U.S. National Library of Medicine, Bethesda, MD), suggesting that a single antibody is unlikely to recognize both PLA_2_R and ADAMTS13. Second, epitope spreading could be considered. It has been reported that epitope spreading can induce an autoimmune disease during chronic inflammation [8]. A cysteine-rich domain has been reported to be the major antigenic epitope in idiopathic MN [9]. C-type lectin-like domain (CTLD)1 and CTLD7 have been newly recognized as antigens by intramolecular epitope spreading [10]. In addition, ADAMTS13 may also be newly recognized as an antigen through intermolecular epitope spreading, producing the ADAMTS13 inhibitor, resulting in the development of acquired TTP. There are some reports of anti-glomerular basement membrane glomerulonephritis and vasculitic glomerulonephritis superimposed on MN [11–13], suggesting that intermolecular epitope spreading could occur in MN by the same mechanism. Lastly, ADAMTS13 expressed on podocytes might become endogenous antigens, and cause MN. ADAMTS13 is mainly expressed in the liver, but is also known to be expressed on the podocytes of glomerular epithelial cells [14], and it may also be a new endogenous antigen for MN.

The anti-PLA_2_R antibody was detected in the serum of our patient. The anti-PLA_2_R antibody test has high sensitivity (82%) and specificity (89%) for idiopathic MN [15]. In a cohort of 131 Japanese patients with MN, there were no false positives for anti-PLA_2_R antibodies [16]. In this case, none of the tests suggested the presence of a collagen disease, viral infection, or malignancy, and the anti-PLA_2_R antibody test was positive and high (89.3 RU/mL). Thus, we considered that idiopathic MN was present at the onset of TTP. Furthermore, ADAMTS13 activity was undetectable and the ADAMTS13 inhibitor was detected. A severe decrease in ADAMTS13 activity (< 0.5%) is more likely in acquired TTP than in secondary TMA [17]. Acquired TTP is fatal if untreated, but can be dramatically improved by plasma exchange therapy [18–20]. Our patient recovered rapidly with plasma exchange therapy, and the remission of TTP has been maintained for several years. Therefore, this case is consistent with acquired TTP.

As mentioned above, 3 cases of the coexistence of idiopathic MN and an acquired TTP have been reported. In the first case, nephrotic syndrome was diagnosed at the onset of the TTP, and a renal biopsy showed characteristic findings of both MN and TMA [6]. However, the anti-PLA_2_R antibody and ADAMTS13 inhibitor were not measured. In the second case, a renal biopsy also showed characteristic findings of MN and TMA, although the case had a history of hepatitis C, with no history of proteinuria [7]. Anti-PLA_2_R antibody and ADAMTS13 inhibitor were detected in the serum, and immunofluorescence staining for renal tissue showed positive for the PLA_2_R and IgG1 subclass, but not for IgG4, whereas in idiopathic MN, IgG4 is usually predominant with immunofluorescence detection of the IgG subclass [21]. It has been reported that PLA_2_R staining in renal biopsies may also be positive for secondary MN which is associated with hepatitis C [22]. In the third case, a patient with frequently relapsing acquired TTP developed nephrotic syndrome 7 years after remission of the TTP, and a renal biopsy showed stage II MN, not TMA. Our case differs from these 3 cases in the following manner. Firstly, primary MN had been proven by renal biopsy prior to the acquired TTP. Secondly, anti-PLA_2_R antibody and ADAMTS13 inhibitor were positive at the onset of the TTP. Lastly, we confirmed that the levels of these 2 antibodies disappeared, and urinary protein levels became undetectable in response to treatment.

A pathological feature of TTP is TMA, and proteinuria is usually detected in TMA. Because TTP is often associated with poor general condition and causes marked decrease in platelets, it is difficult to perform a renal biopsy during the acute phase of TTP. Therefore, even if some cases of TTP are associated with MN, the MN may be overlooked. Therapies for TTP include plasma exchange and steroid therapy, which also reduce anti-PLA_2_R antibody levels as observed in our case [23]. Therefore, the treatment of TTP will ultimately improve the MN because the therapy can reduce the level of ADAMTS13 inhibitor if ADAMTS13 is an antigen for MN. In this case, 3 months after both antibodies’ levels became undetectable, the MN completely resolved, which is consistent with research that states that the disappearance of circulating anti-PLA_2_R antibodies precedes clinical remission by several months in idiopathic MN [24].

In summary, we experienced a patient with idiopathic MN who developed acquired TTP a few years later. Two autoantibodies, the anti-PLA_2_R antibody and ADAMTS13 inhibitor, were detected at the onset of the TTP. We found these antibodies were related to the activity of the TTP and MN in this case. Additionally, there is a possibility that idiopathic MN might be associated with the etiology of some cases of acquired TTP.

## Data Availability

The datasets used and/or analyzed during the current study are available from the corresponding author on reasonable request.

## Abbreviations

MN: Membranous nephropathy
PLA_2_R: Phospholipase A_2_ receptor
THSD7A: Thrombospondin type-I domain-containing 7A
TTP: Thrombotic thrombocytopenic purpura
ADAMTS13: A disintegrin and metalloprotease with thrombospondin type 1 motif 13
PSL: Prednisolone
TMA: Thrombotic microangiopathy
DIC: Disseminated intravascular coagulation
HUS: Hemolytic uremic syndrome; CTLD: C-type lectin-like domain
